# Characterization of functional disability among older adults in Ghana: A multi-level analysis of the study on global ageing and adult health (SAGE) Wave II

**DOI:** 10.1371/journal.pone.0277125

**Published:** 2022-11-03

**Authors:** Kwasi Adjepong Darkwah, Samuel Iddi, Justice Nonvignon, Moses Aikins

**Affiliations:** 1 Department of Epidemiology and Disease Control, School of Public Health, College of Health Sciences, University of Ghana, Legon, Accra, Ghana; 2 Department of Statistics and Actuarial Science, University of Ghana, Legon, Accra, Ghana; 3 Department of Health Policy, Planning & Management, School of Public Health, College of Health Sciences, University of Ghana, Legon, Accra, Ghana; 4 Health Economics, Systems, and Policy Research Group (HESPRG), Department of Health Policy, Planning & Management, School of Public Health, College of Health Sciences, University of Ghana, Legon, Accra, Ghana; McMaster University, CANADA

## Abstract

**Background:**

Functional disability is a common public health problem that affects the health and quality of life of older adults. This causes them to be highly dependent on other members of their family, receive home care, or to be institutionalized. Although functional disability has been widely studied in developed country settings, very limited studies have focused on age-related functional disability in sub-Saharan Africa, and in particular Ghana. The purpose of this study is to assess various factors associated with the difficulties in performing basic and instrumental activities of daily living among older adults in Ghana.

**Methods:**

This cross-sectional study used data on 1610 older adults aged 50 years and above from the Study on Global Ageing and Adult Health (SAGE) survey Wave II conducted in Ghana. Nine standard functioning difficulty tools of WHODAS II was used for the analysis. The WHODAS II offers continuous summary scores with higher scores showing higher disability, and vice versa. A multi-level regression model was used to identify individual and household level risk factors linked to the functional disability of older adults.

**Results:**

Female older adults (53.7%) reported having functional disability. The mean functional disability among older adults aged 50 years and above was 5.2 (± 5.9). Results indicated that older adults who are females, aged 70 years and above, and had three or more chronic conditions had a higher functional disability. Also, older adults who have adequate fruit intake and belong to wealthier households were found to have a lower functional disability.

**Conclusions:**

The study reveals that functional disability among older adults is frequent in Ghana and is associated with having three or more chronic conditions and being overweight/obese. Prevention of functional disability in old age in Ghana is therefore a matter of great social and economic concern, which calls for coordinate efforts across the board to mitigate this public health challenge.

## Introduction

Aging relates to the gradual weakening in physical and psychological activities of the daily living of a person [1, 2]. Thus, disability in performing a basic or instrumental daily activity is the inability of older adults to accomplish a task without support [3]. Generally, disability encompasses impairments, activity limitations, and participation restrictions [4].

Functional disability is a common public health problem that affects the health and quality of life of older adults [5] and causes them to be dependent and often institutionalized [6]. It is also known to be associated with the increasing nursing home admissions, utilization of health care, and the need for family and friends caregivers [7].

Several measures have been used to assess the functional status of the older adults, specifically, the basic Activities of Daily Living (ADL), Instrumental Activities of Daily Living (IADL), and the World Health Organization Disability Assessment Schedule (WHODAS II) [8–10]. Activities of Daily Living are referred to as the activities necessary for an independent life. Examples of ADLs are bathing, dressing, toileting, eating, continence, and transferring. Instrumental Activities of Daily Living refers to activities that require enough ability to make decisions as well as a strong interaction with the environment. Examples of IADL are housekeeping, telephone, shopping, transportation, and managing one’s financial resources and its medication [9, 11]. Instrumental Activities of Daily Living deficiencies mostly precede ADL deficiencies [12]. The WHODAS II scale measures difficulties in activities and daily-life participation [13].

Factors associated with functional disability in older adults have been explored in previous studies using ADL, IADL, and WHODAS scales [8, 10, 14, 15]. Prevalence of functional disability has been identified not to only increase with age [16–18] but relate with gender [18], obesity [19], chronic diseases such as diabetes, stroke, heart diseases [20, 21], rural residence [22] and visual impairments [17].

Although functional disability has been widely studied in developed country settings, there is a paucity of studies examining age-related functional disability in sub-Saharan Africa, and in particular Ghana. Assessing the factors associated with functional disability in this context will help develop appropriate preventive and health care policies and add in the identification of groups of older people at increased risk for functional disability based on these characteristics. Therefore, the purpose of this study is to assess the factors associated with the difficulties in performing basic and instrumental activities of daily living among older adults in Ghana.

## Data description

### Study design

This study was a cross-sectional study which used available data from Global Ageing and Adult Health (SAGE) survey Wave II from 2014 to 2015 in Ghana. SAGE, which is a multi-country study, is conducted in six countries, namely, Ghana, South Africa, India, the Russian Federation, China, and Mexico by WHO to examine the health and well-being of older adults aged 50 years and above and a sample of 18–49 years. The study in Ghana was conducted through a partnership of the World Health Organization (WHO), the Ghana Ministry of Health, and the University of Ghana Medical School.

### Sampling technique

SAGE Wave II adopted a two-stage stratified multistage cluster design. The sample was first stratified by region (all ten administrative regions) across locations (urban/rural). And the second stage selected 30 households from each cluster. Multiple older adults within the same household were included in the SAGE Study.

### Sampling procedure

Face to face interviews with the respondents was conducted by trained data collection teams using standardized questionnaires translated into three local languages; Twi, Akan, and Ga. Some of the data collected include individual and household-level factors such as socio-demographic, chronic health conditions and health services, subjective well-being and quality of life, health care utilization, and preventive health behaviours, and perceived health status, among others. This study is based on data from older adults aged 50 years and above leading to an analytical sample of 1610 respondents after omitting 186 older adults due to missing values for independent variables.

## Measurements

### Functional disability

The dependent variable is the functional disability of older adults aged 50 years and above. The functional disability of older adults was measured using the World Health Organization Disability Assessment Schedule (WHODAS II). SAGE Wave II used nine items found in WHODAS II to measure the level of difficulty confronted in performing basic and instrumental activities of daily living. Out of the 9 items in the WHODAS II scale, 3 items each belong to ADL and IADL. The WHODAS II measure of functional disability among older adults was assessed using a 4-point scale (0 = no difficulty, 1 = mild difficulty, 2 = moderate difficulty, 3 = severe difficulty 4 = extreme difficulty). The scores were summed and range from 0 to 36 with a score < 5 showing the absence functional limitations [23].

### Individual related factors

This study includes a large set of older adults-level variables that are likely to be associated with the functional disability of older adults in Ghana and elsewhere [14, 19, 24, 25]. This includes demographic characteristics of respondents such as age groups (50–59 years, 60–69 years, 70–79 years, 80 years and over) and sex (male, female). Health-related factors such as self-reported chronic conditions were counted and categorized as none, one, two, three, or more. These chronic conditions were stroke, diabetes, angina, hypertension, asthma, chronic lung cancer, oral health, depression, cataracts, and arthritis.

Given the influence of behavioural activities in determining functional disability [26, 27], we include variables such as tobacco smoking status (non-smoker, current daily smoker, occasional smoker, former smoker) and alcohol drinking status (non-drinker, current drinker, former drinker). Nutritional status such as Body Mass Index (BMI) measured in kilogram (kg) per height in squared meters (m^2^), vegetable intake (inadequate intake, adequate intake), and fruit intake (inadequate intake, adequate intake). The grouping of fruit and vegetables into adequate and inadequate was based on the number of servings of fruit or vegetables the participants eat on a typical day. Those who eat less than 5 servings on a typical day were classified as having inadequate fruit or vegetable intake.

The BMI of respondents were classified as underweight (<18.5kg/m^2^), normal (18.5 to 24.9 kg/m^2^), overweight (25 to 29.9 kg/m^2^), and obese (>30 kg/m^2^). The physical activities which were a categorical variable indicating low (<600 metabolic equivalent of task [MET] mins/week), medium (600–3000 MET-mins/day), and high (>3000 MET-mins/week) were constructed from the Global Physical Activity Questionnaire (GPAQ) was used [28].

### Household related factors

We used the wealth index measured as the household’s quintile value on an index of wealth by applying weights to observed household assets using the principal component analysis to control for economic status. The household assets included radio, bicycle, refrigerator, cellular telephone, as well as dwelling characteristics such as type of floor material and wall, and sanitation facilities such as a toilet. The wealth quintiles were distinguished as the poorest, poorer, middle, richer, and richest. We also include other household indicators such as household size (<5 people, ≥5 people), source of water (non-pipe/trunk, pipe/trunk), area of residence (rural, urban), and fuel sources (non-electricity/non-gas, electricity/gas).

## Data analysis

Descriptive statistics of the data are presented using frequencies with their related percentages for categorical variables and summary statistics (mean, standard deviation, minimum, and maximum) for continuous variables. The SAGE Wave II data was observed to have a hierarchical structure of participants nested within survey clusters which could bias the standard errors. Considering the hierarchical structure of the data (older adults nested within households), this study employed a multilevel analysis to accommodate various levels of variability in the data. The multilevel model, which is a regression model, does not only include fixed effects associated with individuals and households, but also random effects that permit specific household-based changes. The model also permitted the inclusion of error terms at each level, which makes it possible to identify changes in variance at each level across models [29]. The random effects can be considered as terms that model other unobserved variables that can affect the functional disability of older adults. Thus, this study uses the random-effects model to estimate the magnitude and significance of clustering (to adjust for dependence) captured through the Intra Class Correlation (ICC). The ICC was computed as the ratio of the variance at the cluster level to the sum of the variances at the individual and cluster levels [30].

The models are specified as follows: Model 1 is a null model that has no predictors but the variance is partitioned into between-individuals and between-household components by adding household-level shifts in the individual-level random intercept value. Model 2 adds predictors at the individual level while maintaining household random intercepts. The predictors at the individual level are sex, age, marital status, highest educational level, number of chronic conditions, physical activity, smoking cigarettes, fruit intake, vegetable intake, and drink alcohol. The log-likelihood ratio test was -4433.8. The intraclass correlation coefficient (ICC) was 0.61, which accounted for 61% of the total variance in the WHODAS at the individual level. The AIC and BIC for model 2 are 8921.7 and 9067.0, respectively.

Model 3 includes only predictors at the household level while maintaining household random intercepts. The predictors at the household level are water, fuel, household size, residence, and wealth quintiles. The log-likelihood ratio test was -5072.0. The intraclass correlation coefficient (ICC) was 0.45, which accounted for 45% of the total variance in the WHODAS at the household level. The AIC and BIC for model 3 are 10166.0 and 10225.2 respectively.

Model 4 is a full model that accounts for individual and household effects while maintaining household random intercepts. The log-likelihood ratio test of -4389.5 and AIC and BIC of 8848.9 and 9037.4, respectively confirm improvement to Model 1 when individual and household predictors are added. The intraclass correlation coefficient (ICC) was 0.61, which accounted for 61% of the total variance in the WHODAS at the individual level. After considering the number of significant variables, the values of the different model selection criteria (AIC and BIC), and the fact that Model 4 has household level significant variables and lower variance of the random effects (which means lower unexplained differences among households), we selected the best fitting model.

All the analyses were performed using STATA version 14. 0 (Stata Corp, College Station, TX, USA) statistical software with model fitting performed using SVY command to account for the complex survey design [31].

Model parameters were obtained using maximum likelihood estimation. To compare different sub-models, we used the Akaike Information Criterion (AIC), Likelihood Ratio Test (LRT), and Bayesian Information Criterion (BIC). Models with lower values of AIC, LRT, or BIC are often preferred. Multicollinearity of the predicted variables was checked using the Variance Inflation Factor (VIF) from multiple linear model, and a VIF value below 10 was considered acceptable [32].

### Ethical requirements

The SAGE Wave II survey was approved by the World Health Organization’s Ethical Review Board (reference number RPC149) and the University of Ghana College of Health Sciences, Ethical and Protocol Review Committee. Hence, this study did not require separate ethics approval. The study participants provided written informed consent.

## Results

Table 1 presents the descriptive statistics of individual and household level characteristics of the study participants. From Table 1, the mean of functional limitations of participants was 5.2 (± 5.9), with a minimum of 0 and a maximum of 36. Out of 1610 participants, 55.4% had no functional limitation. For individual-level factors, 35. 8% of the participants belong to the age group 60–69 years, 53.7% of the participants are females, 51.0% of the participants are married, 43.9% of the participants had no formal education, 65.0% of the participants have no chronic conditions, 51.0% of the participants have high physical activities, 92.7% of the participants do not smoke tobacco, 67.9% do not drink alcohol, 56.8% of the participants have normal BMI, 95.6% of the participants have inadequate fruit intake, and 84.7% of the participants have inadequate vegetable intake. The mean age of participants was 65.8 (± 9.7) years, with a minimum of 50 years and a maximum of 110 years. The mean BMI of participants was 23.5 (± 4.9) kg/m^2^, with a minimum of 13.3 kg/m^2^ and a maximum of 46.3 kg/m^2^. For household-level factors, 62.7% of the participants have their source of water from pipe or trunk water, 88.6% of participants use no-electricity or gas for fuel, 58.8% of the participants have a household size of less than 5, 58.8% of the participants are in the rural residence, and 24.8% of the participants are poorer.

**Table 1:** Descriptive statistics of individual and household-level characteristics of study participants.

Variables	Categories	Frequency (N = 1610)	Percentage (%)
**Dependent Variable**			
Functional Disability Mean (± SD)	5.2 (± 5.9)		
No		892	55.4
Yes		718	44.6
**Independent Variables**			
***Individual Level Characteristics*:**			
Sex:			
	Male	745	46.3
	Female	865	53.7
Age groups, years Mean (± SD):	65.8 (± 9.7)		
	50–59	479	29.8
	60–69	576	35.7
	70–79	401	24.9
	80 and over	154	9.6
Marital Status			
	Unmarried	789	49.0
	Married	821	51.0
Educational Level			
	No formal education	707	43.9
	Primary	201	12.5
	JHS/JSS	217	13.5
	SHS/SSS	191	11.9
	University	223	13.8
	Post graduate degree	71	4.4
Number of Chronic Conditions:			
	None	1047	65.0
	One	390	24.2
	Two	118	7.4
	Three or more	55	3.4
Physical Activity:			
	Low	447	27.8
	Moderate	342	21.2
	High	821	51.0
Smoking Tobacco:			
	Non-smoker	1493	92.7
	Current smoker	64	4.0
	Former smoker	53	3.3
Drink Alcohol:			
	Non-drinker	1093	67.9
	Current drinker	350	21.7
	Former drinker	167	10.4
BMI, kg/m^2^ Mean (±SD):	23.5 (± 4.9)		
	Underweight	207	12.9
	Normal	915	56.8
	Overweight	319	19.8
	Obese	169	10.5
Fruit Intake:			
	Inadequate intake	1539	95.6
	Adequate intake	71	4.4
Vegetable Intake:			
	Inadequate intake	1364	84.7
	Adequate intake	246	15.3
***Household Level Characteristics***:			
Water:			
	Non-Piped/Trunk	600	37.3
	Piped/Trunk	1010	62.7
Fuel:			
	Non-Electricity/Gas	1427	88.6
	Electricity/Gas	183	11.4
Household Size:			
	<5	946	58.8
	≥ 5	664	41.2
Residence:			
	Rural	947	58.8
	Urban	663	41.2
Wealth Quintiles:			
	Poorest	397	24.7
	Poorer	399	24.8
	Middle	311	19.3
	Richer	148	9.2
	Richest	355	22.1

Note: SD = Standard Deviation

Table 2 summarizes the results for four different models considered for the multilevel analysis. The statistically significant fixed effect factors of Model 2 were age, education, smoking cigarette, drinking alcohol, BMI, and fruit intake. The statistically significant fixed effect factors of Model 3 were fuel, residence, and wealth quintile. From the AIC, BIC, and log-likelihood results, Model 4 provides the best fit to the data. The statistically significant fixed effect factors of Model 4 were sex, age, highest level of education, number of chronic conditions, BMI, smoking cigarette/tobacco, drinking alcohol, fruit intake, water, fuel, household size, residence, and wealth quintiles.

**Table 2:** Regression results for two-level models of WHODAS II score.

Variables	Categories	Model 1	Model 2	Model 3	Model 4
Fixed Effects		Coeff. (SE)	Coeff. (SE)	Coeff. (SE)	Coeff.
**Individual Level Factors**					
Sex	Male ^**a**^				
	Female		0.257 (0.224)		0.482 (0.222)^**e**^
Age groups (years)	50–59 ^**a**^				
	60–69		0.636 (0.238) ^**f**^		0.645 (0.232) ^**f**^
	70–79		1.705 (0.271) ^**g**^		1.680 (0.264) ^**g**^
	80 and over		3.833 (0.395) ^**g**^		3.859 (0.388) ^**g**^
Marital Status	Unmarried^**a**^				
	Married		-0.012 (0.226)		-0.252 (0.226)
Educational Level	No formal education ^**a**^				
	Primary		-0.654 (0.311) ^**e**^		-0.302 (0.306)
	JHS/JSS		-0.640 (0.307) ^**e**^		-0.052 (0.308)
	SHS/SSS		-1.063 (0.320) ^**f**^		-0.372 (0.328)
	University		-1.468 (0.306) ^**g**^		-0.770 (0.315) ^**e**^
	Post graduate degree		-1.066 (0.483) ^**e**^		-0.432 (0.485)
Number of Chronic Conditions	None ^**a**^				
	One		0.115 (0.230)		0.215 (0.225)
	Two		0.004 (0.374)		0.333 (0.368)
	Three or more		0.597 (0.528)		1.067 (0.517) ^**e**^
Physical Activity	low ^**a**^				
	moderate		-0.083 (0.279)		-0.213 (0.273)
	High		-0.233 (0.241)		-0.345 (0.236)
Smoking Cigarette/Tobacco	Non-smoker ^**a**^				
	Current smoker		7.252 (0.508) ^**g**^		7.104 (0.495) ^**f**^
	Former smoker		8.139 (0.580) ^**g**^		8.201 (0.566) ^**f**^
Drink Alcohol	Non-drinker ^**a**^				
	Current drinker		0.658 (0.246) ^**f**^		0.602 (0.241) ^**e**^
	Former drinker		0.537 (0.322)		0.480 (0.316)
BMI	Underweight		0.822 (0.296) ^**f**^		0.781 (0.289) ^**f**^
	Normal ^**a**^				
	Overweight		4.333 (0.351) ^**g**^		4.062 (0.343) ^**g**^
	Obese		7.646 (0.422) ^**g**^		7.532 (0.412) ^**g**^
Fruit Intake	Inadequate intake ^**a**^				
	Adequate intake		-2.278 (0.481) ^**g**^		-1.738 (0.482) ^**g**^
Vegetable Intake	Inadequate intake ^**a**^				
	Adequate intake		-0.0565 (0.279)		-1.158 (0.273)
**Household Level Factors**					
Water	Non-Piped/Trunk ^**a**^				
	Piped/Trunk			-0.076 (0.344)	-0.861 (0.230) ^**g**^
Fuel	Non-Electricity/Gas ^**a**^				
	Electricity/Gas			-3.047 (0.480) ^**g**^	-1.072 (0.331) ^**f**^
Household Size	<5 ^**a**^				
	> = 5			0.280 (0.295)	0.534 (0.202) ^**f**^
Residence	Rural ^**a**^				
	Urban			-1.009 (0.332) ^**f**^	-0.460 (0.223) ^**e**^
Wealth Quintiles	Poorest ^**a**^				
	Poorer			-0.830 (0.416) ^**e**^	-0.778 (0.395) ^**f**^
	Middle			-1.902 (0.460) ^**g**^	-1.197 (0.282) ^**g**^
	Richer			-1.440 (0.592) ^**e**^	-0.665 (0.314)
	Richest			-1.155 (0.489) ^**e**^	-0.679 (0.403)
Constant		5.162 (0.148) ^**g**^	1.869 (0.455) ^**g**^	6.814 (0.346) ^**g**^	2.930 (0.478) ^**g**^
**Random Effects**					
Log-Likelihood		-5127.5	-4433.8	-5072.0	-4389.5
AIC/BIC		10261.0/10277.2	8921.7/9067.0	10166.0/10225.2	8848.9/9037.4
The variance of random effect		16.72	8.95	14.52	8.40
Variance of residuals		17.72	5.68	17.66	5.44
Degrees of freedom of the residual		1607	1583	1599	1581
Household Level ICC		0.49	0.61	0.45	0.61

Note. Coeff. = Coefficient, SE = Standard Error

^a^ Reference group

^b^ LR: Likelihood Ratio

^c^ ICC: Intraclass correlation coefficient

^d^ AIC/BIC: Akaike’s Information Criterion/ Bayesian Information Criterion

^e^ P<0.05

^f^ P<0.01

^g^ P<0.001

Female older adults have a higher functional disability compared to male older adults, which was statistically significant (Coefficient (*β*) = 0.482, SE = Standard Error (SE) = 0.222). Older adults who belonged to the age group 60–69 years (*β* = 0.645, SE = 0.232), 70–79 years (*β* = 1.680, SE = 0.264), and 80 years and above (*β* = 3.859, SE = 0.388) had higher functional disabilities compared to older adults who belonged to the age group 50–59 years. Older adults who have completed university had lower functional disability compared to older adults who had no formal education (*β* = −0.770, SE = 0.315).

Older adults who have three or more chronic conditions present had higher functional disability compared to older adults who had no chronic condition present (*β* = 1.067, SE = 0.517).

Older adults who are current smokers (*β* = 7.104, SE = 0.495) and former smokers (*β* = 8.201, SE = 0.566) of cigarette/tobacco had higher functional disability compared to older adults who are non-smokers of cigarette/tobacco. Older adults who are current alcoholic drinkers had higher functional disability compared to older adults who are non-alcoholic drinkers (*β* = 0.602, SE = 0.241).

Older adults who are underweight (*β* = 0.781, SE = 0.289), overweight (*β* = 4.062, SE = 0.343), and obese (*β* = −7.532, SE = 0.412) had higher functional disability compared to older adults who had normal weight.

Older adults who have adequate fruit intake had lower functional disability compared to older adults who had inadequate fruit intake (*β* = −1.738, SE = 0.482). Older adults who used piped/trunk water had lower functional disability compared to older adults who do not use piped/trunk water (*β* = −0.861, SE = 0.230). Older adults who used electricity/gas had lower functional disability compared to older adults who do not use electricity/gas (*β* = −1.072, SE = 0.331). Older adults whose household size is greater than or equal to 5 had functional disability compared to older adults whose household size is less than 5 (*β* = 0.534, SE = 0.202).

Older adults who lived in urban residence had lower functional disability compared to older adults who lived in rural residence (*β* = −0.460, SE = 0.223). Older adults who were classified as poorer (*β* = −0.778, SE = 0.395) and middle wealth quintile (*β* = −1.197, SE = 0.282) had lower functional disability compared to older adults who were classified as poorest.

## Discussion

Functional disability is a major health problem among older adults worldwide because of their inability to do daily activities, significantly affecting their quality of life [33]. Older adults who had functional disability are at increased risk for institutionalization, hospitalization, and death [34]. There are various determinants of functional disability and in diverse settings [35].

The findings from this study suggest that individual and household level characteristics matter in explaining the functional disability of older adults in Ghana. For individual-level effects, this study found that older female adults are more commonly to have higher functional disability scores as compared to older male adults. These findings are consistent with other studies [25, 36–38]. The high functional disability in women maybe due to their higher life expectancies [39, 40]. Also, this study found that functional disability scores increases with age especially in the oldest old people and this is consistent with studies [14, 25, 36, 37, 41, 42]. Age is identified to be related to functional disability because health problems, comorbidities such as disabilities, hypertension, and disabilities increase with age [37, 43].

Furthermore, the study found that older adults who have completed tertiary education are more commonly to have less functional disability scores compared with older adults with no formal education, similar to other studies [14, 41]. According to Goswami et al. [14] and Sinalkar et al. [41], educated older adults may have higher health literacy, maintain a better IADL performance, and maintain their physical function with a positive attitude.

Moreover, the study identified that older adults with three or more chronic conditions are more commonly to have higher functional disability scores compared with older adults with no chronic condition which is similar to a study by Ralph et al. [44]. According to Goswami et al. [14], older adults are more prone to communicable and non-communicable diseases which result in functional disability score.

The study found that older adults who have adequate fruit intake are more commonly to have less functional disability scores compared with older adults who have inadequate fruit intake which is similar to a study by Alipanah et al. [27]. According to Nicklett and Kadell [26], fruit intake protects older adults against the development of functional limitations and chronic conditions.

This study found that older adults who smoke tobacco or drink alcohol are more commonly to have higher functional disability scores compared with older adults who do not smoke tobacco or drink alcohol which is similar to the findings of Artaud et al. [45]. Smoking tobacco and alcohol intake increases the functional disability of older adults which makes them have long years in ill health [46–48].

However, the study found that physical activities was not associated with functional disability of older adults which is contrary to a study by Ćwirlej-Sozańska et al. [9]. Regular physical activity such as low intensity walking to more vigorous sports aids in physical and mental functions and the decrease of some effects of chronic diseases such as muscular weakness [49, 50].

For household-level effects, this study found that older adults who have household size more than five are more commonly to have less functional disability scores compared with older adults who have a household size less than five which is similar to other studies [51]. According to Saito et al. [52], the functional disability increases among older adults living alone and those living only with a spouse. This is because, older adults with few household members are unable to get support to carry out their functional abilities such as walking, dressing. This results in psychological disorders such as depression in older adults [53, 54].

Also, this study found that older adults who reside in urban areas are more commonly to have less functional disability scores compared with older adults who reside in rural areas which is similar to other studies [55, 56]. In Ghana, most of the occupation in the rural areas are farming activities which involve more physical activities and also, there are fewer activities in the rural areas that involve the use of buses or cars as means of transportation. Laditka et al. [57] stated that older adults in rural areas experience a longer expected period of disability in performing basic activities of daily living than older adults in urban areas. The higher rate of disabilities among older adults residing in rural areas may be due to less education, less access to health care and preventive services, pooer living conditions, and poverty [55]. Furthermore, this study found that older adults who drink pipe or trunk water are more commonly to have less functional disability scores compared with older adults who do not drink pipe or trunk water. Thomas et al. [58] stated that older adults are susceptible to dehydration as a result of acute and chronic health problems such as functional disability, which worsen thirst, decrease the ability to drink adequately, and increase the loss of skin, urinary, and respiratory fluid. Hence, older adults developing the habit of drinking water from the pipe helps them not to be dehydrated.

Finally, this study found that functional disability scores decreased among older adults who belong to the second to fifth wealth quintile. According to Wahrendorf et al. [59], there is a high disability among older adults with low wealth compared to those with high wealth. Poor older adults are unable to afford better healthcare when they are experiencing functional disability. Elwan [60] stated that conditions related to poverty such as inadequate water and sanitation, lack of access to healthcare, and poor living conditions increase the risk of disabilities in low- and middle-income countries. Nevertheless, it is easier for wealthy families to get caregivers to assist older adults with functional disabilities [61].

This study adds to the body of knowledge on aging and functional disability in a developing country setting such as Ghana. This paper makes three scholarly contributions. First, in the context of a developing nation such as Ghana, this study assesses the influence of individual-level factors such as performing physical activities, vegetable and fruit intake, and household level factors such as water, household size, and wealth quantile on functional disability with special emphasis on the older adults. The multilevel individual and household effects make the findings fascinating and the implications to the policy very vital.

Second, this study finds factors like fruit intake that may reduce functional disability among older adults. Thus, our findings highlights the need for nutritional interventions such as a high intake of fruits to reduce the functional disability among older adults. This is crucial right now because diets high in sugar, fat, and animal-based protein and sedentary lifestyle changes in developing countries make older adults more vulnerable to obesity, which has an impact on the functional disability. Finally, functional disability among older adults in Ghana presents a number of health risks, making it a public health issue that must be addressed with the appropriate policies, awareness campaigns, and interventions.

There were some limitations. This study is cross-sectional, thus, causal inference is limited. Rigorous designs such as longitudinal or randomized controlled trial with a strong qualitative data to better understand the relationship between individual and household related factors and functional disability of older adults.

## Conclusion

The influence of individual and household level factors on the functional disability of older adults in Ghana is real. Functional disability among older adults is frequent and not only lowers the quality of life of the older adults but strains society’s limited resources for assistance, care, and rehabilitation. With the increasing population of the older adults in Ghana, there is an urgent need for the development of national policy on physical activity and nutritional interventions for the older adults which is currently not emphasized in the Nation Health Policy.
